# Editorial: On the “Human” in Human-Artificial Intelligence Interaction

**DOI:** 10.3389/fpsyg.2021.808995

**Published:** 2021-12-24

**Authors:** Stefano Triberti, Ilaria Durosini, Jianyi Lin, Davide La Torre, Manuel Ruiz Galán

**Affiliations:** ^1^Department of Oncology and Hemato-Oncology, University of Milan, Milan, Italy; ^2^Applied Research Division for Cognitive and Psychological Science, IEO, European Institute of Oncology IRCCS, Milan, Italy; ^3^Dipartimento di Scienze Statistiche, Università Cattolica del Sacro Cuore, Milan, Italy; ^4^Department of Mathematics, Khalifa University of Science and Technology, Abu Dhabi, United Arab Emirates; ^5^SKEMA Business School and Université Cote d'Azur, Sophia Antipolis Campus, Sophia Antipolis, France; ^6^Department of Applied Mathematics, University of Granada, Granada, Spain

**Keywords:** artificial intelligence, eXplainable Artificial Intelligence (XAI), human centered AI, human technology interaction, cyberpsychology, attitudes toward technology

Artificial Intelligence or technologies able to perform tasks normally requiring human cognitive processes (e.g., reasoning, perception) are revolutionizing many fields such as healthcare and business. For example, medical doctors use artificial intelligence to analyze pathological data and patients' genomic profiles to identify personalized treatment according to a precision medicine approach. In general, artificial intelligence represents an invaluable resource for any professional dealing with the need to understand data and make decisions.

However, desirable utilization of technology largely depends on the interface that allows users to form a representation of software's structure and functions. Research is still needed to provide information on how humans represent artificial intelligence. This is important especially when the future users are not experts in algorithms but they still need to make decisions based on deep learning outcomes. Last but not least, we still have to understand and master the multiple ways artificial intelligence could be used to address *human* issues: how can artificial intelligence contribute to improving people's health, well-being and flourishing?

Psycho-social research shows that technologies are not accepted by users and implemented in real-life on the sole basis of effectiveness. People form attitudes toward technologies that shape their future behavior (Venkatesh and Davis, 2000; Marangunć and Granić, 2015; Gorini et al., 2018; Nunes et al., 2019); or, they evaluate technologies according to pre-existing intentions, needs and misconceptions that may lead to improper usage, errors, and ultimately abandonment (Triberti et al., 2016; Sebri et al., 2020). Without an understanding of the human barriers and motivations for adoption and acceptance of AI, AI is simply just an invention in search of a market.

To understand human responses to AI, we identify five categories of potential scientific areas requiring further investigation for this special issue:

- The study of attitudes and behaviors toward artificial intelligence (Dos Santos et al., 2019; Schepman and Rodway, 2020; Sebri et al., 2020) (area A);
- The study, development, and validation of artificial intelligence-human interfaces; this includes eXplainable Artificial Intelligence (XAI), or the sub-discipline devoted to make “black-box” algorithms understandable to human users (Miller, 2019), and Human Factors research on systems involving artificial intelligence (Knijnenburg et al., 2012; Lau et al., 2020) (area B);
- The research on human characteristics that could hinder or promote effective interaction with artificial intelligence (Oksanen et al., 2020; Sharan and Romano, 2020; Matthews et al., 2021); this includes models and criteria to select personnel expected to work with artificial intelligence (La Torre et al., 2021) (area C);
- The identification of issues in artificial intelligence implementation and/or possible solutions to existing issues, including social science, political science, and philosophy/ethics contributions (Pravettoni et al., 2015; Triberti et al., 2020a,b) (area D);
- Research on the implementation or testing of specific artificial intelligence solutions that require interaction with human users, and provides information relevant to better understand risks and opportunities (Adamo et al., 2015; Bodini et al., 2018) (area E).

The present special issue aimed at collecting innovative and interdisciplinary contributions on the topic of artificial intelligence-human interaction, that emphasize the “human” part and provide insights to improve the development of artificial intelligence that could be really useful and effectively used in society. All the contributions to this special issue indeed touch on one or more of the research areas highlighted above, as it is evidenced below by reference to the designated areas' letters.

Specifically, the contribution by Biancardi et al. (areas A, B, C) deals with the topic of interface, specifically in terms of embodied conversational agents: it elaborates on the topic of adaptation, testing three different models that allow embodied conversational agents to modify their behavior based on the user's response. They show that the way we conceptualize adaptive interfaces affects users' engagement with artificial intelligence.

In this line, the theoretical contribution by Hildt (areas A, B, D) reflects on how humans would like to interact with robots and how the interaction influences both parts. It is suggested that a broader perspective on Human-Robot Interaction is needed that takes the social and ethical implications into account. Although humans tend to react to robots in similar ways as they react to human beings even if they are not, aspects needing more attention include how to deal with simulated human-like behavior that is not grounded in human-like capabilities. Moreover, questions of what social roles to ascribe to robots deserve a central importance in designing them.

Interface and its ethical and practical aspects are elaborated further in the contribution by Holohan and Fiske, dealing mostly with area D, focused on artificial intelligence in psychotherapy and the concept of transference: indeed both these studies show that we may need to update conceptions, theoretical constructs, and terminology to support desirable implementation of artificial intelligence solutions within sensitive contexts, such as healthcare. Design thinking and the associated research methods may be an important resource to conceptualize artificial intelligence solutions that address real-world issues, as suggested by the perspective article by Talamo et al. (area B) focused on systems to support venture capitalists' decision-making. Indeed, one possible way to improve artificial intelligence is to consider users' needs and context since the first steps of the design of both algorithms and interface, consistently with a user-centered approach (Weller, 2019). From a broader point of view, the two reviews by Tariq, Poulin et al. (areas A, C, D) and Abonamah et al. (area D) also help to identify relevant factors involved both in operational excellence and commoditization of artificial intelligence. In particular, the former sheds novel light on how artificial intelligence can provide driving forces for achieving operational excellence in a business company (Gólcher-Barguil et al., 2019) as soon as certain barriers consisting of lack of skills, technologies and strategy can be overcome, while the latter well-interprets and outlines the role of artificial intelligence technologies as commodities within an organization in a comprehensive and systematic way comparing to existing literature (Carr, 2003).

Furthermore, it is important to take into account all psychological, medico-legal, and ethical issues which need to be addressed to artificial intelligence be considered fully capable of patient management in real life. Coppola et al. (areas C and D) provide an overview of the state of the art of artificial intelligence systems regarding medical imaging, with special focus on how artificial intelligence can be implemented in a human-centered field such as contemporary medicine. This approach contributes in addressing important issues associated with artificial intelligence in sensitive contexts (e.g., ethical and organizational) (Keskinbora, 2019; Triberti et al., 2020a), as it encourages health professionals to actively engage in iterative discourse to preserve humanitarian sensitivity in the future models of care.

Tariq, Babar et al. related to category E, propose and test a framework based on Apache Spark for efficiently processing the big datasets resulting from user comment activities triggered by videos on social media. The article shows the potential effectiveness of the devised implementation, which was able to perform the planned analytics operations on social media dataset in a time that well-scales with the data size. Specifically, they provide a new concrete demonstration of processing big data coming from an extended social hub named Dailymotion within a time frame of few minutes using Apache Spark.

Certainly future research needs innovative tools and approaches to address human behavior through the lenses of artificial intelligence. An example of integration between artificial intelligence and social psychology methods is the work by Catellani et al. (area E) who, moving from the psychological concept of framing, test persuasive messages to do home-based physical activities and use the results to inform the development of a Dynamic Bayesian Network predictor. This points toward the development of artificial intelligence-based tools that autonomously interact with human users to support positive behavioral change. Similarly, Peifer et al. (area E, possibly with interesting hints for future research in areas B and C too) focus on team flow (i.e., a shared experience characterized by the pleasant feeling of absorption in challenging activities and of optimal team-interaction during an interdependent task), a well-known concept in group and work psychology. They identify psychophysiological and behavioral correlates which can be used as input data for a machine learning system to assess team flow in real time. Such approaches constitute notable examples of how artificial intelligence could provide new avenues for research and intervention on human behavior, consistently with the prediction that artificial intelligence will play a more and more important role in psychological research (Lisetti and Schiano, 2000; Daróczy, 2010; Tuena et al., 2020).

In conclusion, this Research Topic provides an overview on artificial intelligence-human interaction, focusing on relevant psychological, technical, and methodological aspects of real-life implementation. Emphasizing the “human” in the human-artificial intelligence interaction provides insights to design the future technologies that could contribute to advance society.

## Author Contributions

ST and ID drafted the editorial. JL, DL, and MR participated in the discussion on the ideas presented and edited the editorial. All authors approved the submitted version.

## Funding

ST was supported by MIUR—Italian Ministry of University and Research (Department of Excellence Italian Law n.232, 11th December 2016) for University of Milan. ID was supported by Fondazione Umberto Veronesi.

## Conflict of Interest

The authors declare that the research was conducted in the absence of any commercial or financial relationships that could be construed as a potential conflict of interest.

## Publisher's Note

All claims expressed in this article are solely those of the authors and do not necessarily represent those of their affiliated organizations, or those of the publisher, the editors and the reviewers. Any product that may be evaluated in this article, or claim that may be made by its manufacturer, is not guaranteed or endorsed by the publisher.
